# Efficacy of brexanolone in postpartum depression

**DOI:** 10.1192/j.eurpsy.2023.1751

**Published:** 2023-07-19

**Authors:** C. M. Rodríguez Mercado

**Affiliations:** Unidad de Tratamiento de Toxicomanías. Mieres, Servicio de Salud Del Principado de Asturias, Mieres, Spain

## Abstract

**Introduction:**

Depression whose onset in the peripartum period is commonly referred to as Postpartum Depression.Postpartum Depression affects 11.5% of women during pregnancy or postpartum.Brexanolone has been developed for the treatment of Postpartum Depression, it is a formulation of Allopregnanolone, which is administered parenterally. Several studies have found that a single Brexanolone infusion had rapid antidepressant effects for severe Postpartum Depression, with good safety.

**Objectives:**

The objective of this review is to evaluate the effectiveness of treatment with Brexanolone in Postpartum Depression.

**Methods:**

A systematic search was performed through Pubmed, including randomized controlled trials (RCTs).

**Results:**

2 articles were selected that included three RCTs, in which participated 156 women diagnosed with Postpartum Depression who received an infusion of Brexanolone and 111 women with Postpartum Depression who received placebo .Compared to placebo, women who received Brexanolone had a significant clinical response, starting 24 hours after administration (RR: 1.34; 95% CI: 1.03–1.73). ), reaching its maximum action peak at 36 hours ( RR: 1.50; 95% CI 1.06–2.13; P = 0.02 ), and with a duration of effect up to the seventh day ( RR = 1.32, 95% CI: 1.01–1.73). Likewise, women with Postpartum Depression, treated with Brexanolone, had a significantly greater remission, starting at 24 hours of treatment (RR: 1.86; 95% CI: 1.03–3.34), reaching its peak maximum action at 60 hours ( RR: 2.20; 95% CI 1.31–3.70 ), and with a duration of effect up to 72 hours ( RR = 1.96; 95% CI: 1 .41–2.72).

**Conclusions:**

The administration of a dose of Brexanolone seems to have an ultra-rapid antidepressant effect for the treatment of Postpartum Depression. The short-term and long-term therapeutic effect of brexanolone needs to be examined with large-scale randomized controlled studies.

**Disclosure of Interest:**

None Declared

